# Evaluation of the antidyslipidemic and nephroprotective effect of methanolic seed extract of *Lepidium sativum* on male Swiss albino mice fed on deep fried palm oil

**DOI:** 10.3389/fnut.2025.1468704

**Published:** 2025-07-11

**Authors:** Ebsa Tofik Ahmed, Abebe Dukessa, Tigist Mateos, Welela Meka, Meskelu Seyoum Moti, Belay Zawdie

**Affiliations:** ^1^Department of Medical Biochemistry, School of Medicine, College of Health and Medical Sciences, Haramaya University, Harar, Ethiopia; ^2^Department of Biomedical Sciences, College of Health Sciences, Jimma University, Jimma, Ethiopia; ^3^Department of Chemistry, College of Natural and Computational Sciences, Mattu, Ethiopia; ^4^School of Medicine, Institute of Health Sciences, Wallaga University, Wallaga, Ethiopia

**Keywords:** *Lepidium sativum*, palm oil, kidney, lipid profile, histopathology

## Abstract

**Introduction:**

Currently, there is dramatic change in dietary habits. Consumption of energy dense foods are becoming common practice globally. Increased consumption of energy dense foods are main cause of disorder on lipid metabolism and kidney function, which are primary risk factors for many chronic diseases. Thus, this study aims to evaluate antidyslipidemic and nephroprotective effect of methanolic seed extract of *Lepidium sativum* (MSELS) on mice fed on deep fried palm oil.

**Methods:**

The study carried out using 24 mice that grouped into four groups. G-I fed on normal mice pellets and distilled water. G- II fed on deep fried palm oil and distilled water. G-III and IV fed on deep fried palm oil and treated with MSELS of 200 and 400 mg/ kg/day, respectively. Each group treated per orally for 8 weeks. At the end of study, mice fasted overnight, anesthetized and blood taken by cardiac puncture for lipid profile and kidney parameters. Then, sacrificed by cervical dislocation, liver and kidney tissues taken for histopathology investigation.

**Result and Discussion:**

The serum total cholesterol (TC), low density lipoprotein cholesterol (LDL-C), and triglyceride (TG) levels decreased while high density lipoprotein cholesterol (HDL-C) increased significantly in G- IV only whereas serum LDL-C/HDL-C ratio and creatinine levels showed a significant decrement in both G- III and IV when compared with G- II. The serum blood urea nitrogen (BUN) and uric acid levels decreased in G- III and IV even though only serum uric acid value in G-IV decreased significantly when compared with G-II. Besides, G-IV showed significant reduction in liver weight as well as restoration of liver and kidney histopathology when compared with G- II than G-III.

**Conclusion:**

Based on the above results, MSELS showed better antidyslipidemic and nephroprotective effect on male mice treated with deep fried palm oil at the dose of 400 mg/kg/day of MSELS.

## Introduction

Palm oil is one of the edible vegetable oil which obtained from the mesocarp fruits of the tropical *Elaeis guineensis*. It constitutes around one-third of global vegetable oil consumption and three- quarters of which is used for food. It is consumption has increased by tenfold as it represents 30% of the world’s vegetable oil production (1). Fresh palm oil contains almost 50% saturated fatty acids (SFA), 50% monounsaturated fatty acids (MUFA) and low levels of polyunsaturated fatty acids (PUFA) (2). Besides, it contains carotenoids, tocopherols and tocotrienols antioxidants. The antioxidant effect of tocotrienols relative to tocopherols is 50 times higher than that of tocopherols. These chemical compositions which intensify its oxidative stability and low in price among dietary oils enhance high percentages of palm oil to use for frying (3, 4).

Frying is one of the most common methods of food preparation worldwide. During the frying process, the oil continuously exposed to a high temperature in the presence of air and food moisture. Frying methods vary based on the amounts of oil used, the way of handling food and cooking times (5). Deep-frying is one of the most widely used frying methods. It involves immersing food completely in hot oil with contact among oil, air and food moisture at a temperature of 150 to 200°C (6).

Deep-frying is one of the most common methods of food preparation worldwide. It is not only limited to homes, but also practiced regularly in among street food vendors, restaurants and commercial food industry. These are due to its operational simplicity, economic affordability, and ability to improve sensory characteristics in fried foods that are highly appreciated by the consumer (7, 8). However, as the method of deep-frying requires a huge amount of oils people most often keep the used frying oil for reuse to enable cost-effectiveness. Besides, the main reasons are due to low level of awareness among the large community about its hazardous effect on health (9).

After consumption of fried foods, some of the formed harmful products enter into the gastrointestinal tract (GIT) and blood circulation where it affects lipid metabolism and kidney function through their own mechanisms. As supported by prior clinical studies, some of the formed products can increase LDL-C level which, is one of the most common and major dyslipidemia type that are risk factor for atherosclerotic CVDs. They increase LDL-C level through either of the following mechanism such as increasing the production rate of VLDL-C, increased VLDL-C to LDL-C conversion as well as reduced LDL-C clearance by decreasing the expression of LDL-C receptors (10). For example as supported by previous study, the polar compound formed during the deep-frying of oil can elevate the expression level of microsomal triglyceride transfer protein (MTP) (11).

The MTP play a critical role in the assembly and secretion of apoB-containing lipoproteins such as VLDL-C. The increased expression levels of MTP results in increased secretion of VLDL-C, together with the downstream effects on HDL-C and LDL-C in a way that triggers atherogenic effect (12, 13).

Not only the polar compound contribute for the increment of LDL-C or other lipid parameters in a direction they can induce atherosclerotic CVDs but also the saturated fatty acid, trans fat can increase especially the LDL-C through decreasing the clearance and by increasing the production rate of LDL-C from VLDL-C (14).

The elevated LDL-C, by the formed different harmful products, oxidized easily from the circulating lipids because of their high PUFA content and the oxidatively modified LDL-C more avidly taken up by macrophages via the scavenger receptor than native LDL-C. These cause deposition of lipid in the endothelial space that leads to atherosclerosis which are primary risk factors for cardiovascular disease (CVDs) (15, 16).

Even though there are many synthetic drugs on the market for the clinical treatments of dyslipidemia, the statins are widely used drugs because of its significant effectiveness in lowering the level of LDL-C. However, statin consumption results in adverse effects such as liver damage, muscle toxicity, myopathy and acute kidney failure. Furthermore, the use of statin drugs along other drugs either for treatment of liver or kidney damage can elevate the extent of organ damage rather than its curative effect (17, 18).

Besides, the deep frying process resulted in the formation of many toxic oxidative products that damage internal organ like kidney. Furthermore, as reported in a previous study, consumption of heated oil causes increased oxidative stress, which leads to kidney damage manifested as altered kidney biochemical parameters and damaged kidney histopathology (19, 20). A previous study (19) established that consuming fried oil for results in abnormal changes in kidney biochemical parameters and kidney histopathology.

Even though many drugs are available for kidney diseases, they do not give satisfactory results due to adverse effects and high cost. Thus, the side effects of synthetic drugs and their high cost in treating kidney diseases increased the need to look for herbal medicine that are in the form of dietary supplementation and rich in different important phytoconstituents that capable of restoring kidney biomarkers and kidney histopathology to normal through mitigation of the products from repeatedly deep frying of palm oils. As a result, it’s critical to create alternate techniques for reducing the detrimental health impacts of often consuming deep-fried palm oil that are less dangerous, more affordable, readily accessible locally, and easy to use. *Lepidium sativum* is one of the most important vegetables and herbal medicines. In Ethiopia, there are traditionally claims on *L. sativum* seed as it used to treat hypertension, liver diseases and kidney diseases even though this use is not more scientifically reported.

Furthermore, its seed is one of the functional foods and that contains ingredient such as saponin, flavonoids, alkaloids, terpenoids and steroids which have antioxidant, antiatherosclerotic, reno-protective, antidyslipidemic and hepatoprotective capacity as supported by different literature (21). The previous study reported the concentrations of six major flavonoids in *L. sativum* L. seed as follows naringin (12.4 mg/g), quercetin (2.81 mg/g), naringenin (24.87 mg/g), luteolin (2.34 mg/g), kaempferol (1.82 mg/g), and apigenin (0.95 mg/g) (22).

For example,: the previous study elucidated as naringenin action primarily affects lipid metabolism though down-regulating the expression of genes related to fatty acid biosynthesis such as fatty acid synthase (FAS), acetyl-coA carboxylase (ACC), 3-hydroxy-3-methylglutaryl-coenzyme A reductase (HMGCR), and sterol regulatory element-binding protein 1c (SREBP-1C) while up-regulating the expression of gene associated with fatty acid oxidation like carnitine O-palmitoyl transferase 1 (*CPT-1*) gene as revealed in previous research that involves *in vivo* and *in vitro* techniques (23).

Kaempferol contributes to the prevention of atherosclerosis by boosting the hepatic expression of the low density lipoprotein cholesterol receptor (LDL-C-R). The previous experimental study revealed as kaempferol decreased fat weight, serum TC, LDL-C; and HDL-C (24). Besides, naringin which has anti-inflammatory and potential free radical scavenging effects inhibits glomerular dysfunction and renal injury by triggering the nuclear factor erythroid 2-related factor 2 (Nrf-2) pathway and declined pro inflammatory factors such as cyclooxygenase-2 (COX-2), tumor necrosis factor-α (TNF α), inducible nitric oxide synthase (iNOS), and apoptosis agents (25). Thus, the *L. sativum* seed might poses good therapeutic value due to its composition of different flavonoid classes. Therefore, this study aimed to evaluate antidyslipidemic and nephroprotective effect of MSELS on mice fed on deep fried palm oil.

## Materials and methods

### Study setting and study design

Experimental study design done for 8 weeks/2020 on Male Mice at Veterinary medicine post graduate laboratory of Jimma University.

### Experimental animals

Twenty-four male Swiss albino mice were used in this that weighing 30–35 g and aged 8 to 10 weeks. Mice taken from the Tropical and Infectious Disease Research Center (TIDRC), Sokoru, Jimma, Southwestern Ethiopia. Then, brought to Veterinary Medicine Postgraduate Laboratory and had free access to normal mice pellet and distilled water in accordance to the National Institutes of Health (NIH) Guidelines for Care and Use of Laboratory Animals (26). The mice housed in a transparent plastic cage and with SS sipper 250 mL water bottle at room temperature of 20–26°C, relative humidity of 40–50% and 12 h light/dark cycle. The mice acclimatized to the laboratory environment for 14 days before subjected to the experiments.

### Animal protocol

The mice divided randomly into four groups that contain 6 mice per cage. Each mouse in each group identified from one another by making a number label on their tail by using a permanent marker. During the commencement of the experiment and then per week, mouse body weight measured by using balance to adjust the MSELS dose level administration. Acute oral toxicity (limit) test was carried out on female mice according to OECD’s guideline (27). The mice were administered via oral gavage at doses of 2,000 mg/kg body weight/day. The mice were frequently observed for behavioral changes and common toxicity signs after dosing for the first 24 h and observation was continued daily for two consecutive weeks. No sign of toxicity or mortality was observed within 24 h as well as over a given 2 weeks. Therefore, based on this and OECD’s guideline protocol, the first extract dose was 10% of 2000 mg/kg body weight (tested limit dose) and the second dose was scaled up by doubling the initial dose (Table 1).

**Table 1:** Animal protocol.

Groups	Dose administration
I	Normal Mice Pellets + Distilled Water
II	Fried Palm Oil (FPO) + Distilled Water
III	FPO + 200 mg/kg/day MSELS
IV	FPO + 400 mg/kg/day MSELS

The letter indicates: FPO = Fried Palm Oil, MSELS = Methanolic Seed Extract of *Lepidium sativum*.

### Plant material preparation

The plant material preparation conducted in Organic Chemistry laboratory, Natural Science College, Jimma University. The seeds winnowed, washed, shade dried and ground into coarse powder using mortar and pestle. The coarse powder weighed and then packed well in a clean plastic container to avoid the entrance of air and other surrounding material until extracted.

### Preliminary phytochemical screening

Different organic solvent extracts of *L. sativum* seed used to screen the following phytochemicals like alkaloid, phenolic compound, flavonoid, saponin, steroid, terpenoid, and quinone. The extract of the methanol, chloroform, hexane and acetone was prepared by dissolving the 15 g seed powder in 50 mL beaker separately for each solvent. The methods of screening employed were those described by Ahmed et al. (28) for the presence of various active components.

### Preparation of plant material extract

The coarse seed powder (400 g) extracted by maceration in methanol for 72 h at room temperature by shaking three times per day throughout the maceration time. The mixture first filtered using cotton wool and then with Whatman No. 1 filters paper. The residue re-macerated for another 72 h twice and filtered. The combined filtrate separated by rotary evaporator at 50°C and 90 rpm.

Then, the filtrate taken to the thermostatic oven at 40°C and kept overnight to evaporate the remaining methanol. Then, the total dried extract harvested and kept in a desiccator to maintain dryness throughout the experimental period (29, 30).

% yield $=\frac{\text{weight of the dried extract}}{\text{weight of the seed powder}}$ ×100.

### Preparation of fried palm oil

The preparation of fried palm oil was carried out according to the previously described methods of Sunarti et al. (31) and Famurewa et al. (32) which fried three and five times with minor modification based on information gathered from street food vendors in Jimma (study area). Potatoes and palm oil purchased from the market, Jimma, Ethiopia. Potato washed, peeled and cut into slices of uniform size using a vegetable slicer. The sliced potatoes kept in water, blotted with tissue paper and then, 500 g weighed for the frying process. Then, 2.5 L of fresh palm oil added into a deep fat fryer and frying carried out at a temperature of 200°C. A batch of 500 g raw sliced potatoes fried for 20 min and then, the fried potatoes batch removed from the fryer. Then, the frying operation carried out for a new potato batch. The frying procedure was done once daily for five consecutive days. The same oil used repeatedly to fry the next batch of potatoes without adding any fresh palm oil to top up the lost oil during frying process. At the end of the frying, oil taken out, filtered, kept in a bottle until used for further experimental procedure.

### Administration of fried palm oil

As most of the time edible oils consumed along the foods, the fried palm oil of the current study also provided along the normal mice pellets. Besides, the level of palm oil in most African dishes is approximately around 15% (33). Accordingly, the animal diet was prepared by mixing fried palm oil with normal mice pellets to contain 15% fried palm oil. The normal mice pellets diet of 85% w/w mixed manually with prepared fried palm oil of 15% w/w. The mixtures left to absorb the fried palm oils at room temperature overnight before the feeding was done (34, 35).

### Data collection

The body weight of the mice taken at the interval of a week to observe body weight change in all groups of animals during the study period. At the end of the study, mice in all groups were fasted overnight and anesthetized with 100 mg/kg ketamine/12.5 mg/kg xylazine. Then, 1.8–2.5 mL of the blood taken from each mouse through cardiac puncture and sacrificed by cervical dislocation. Then, the blood collected with the serum separator tube (SST) and left for 30 min at room temperature to clot. The serum separated through centrifugation with speed of 3,000 rpm at room temperature for 10 min, Finally, the serum were put in the refrigerator at −20°C until it was analyzed for the lipid profiles and kidney function tests. In addition, for histopathology study liver and kidney tissues taken from each mouse carefully after the mice were sacrificed and dissection from the neck to the pubis using sterile surgical blade.

### Histopathology

A mouse of each group was sacrificed following 100 mg/kg ketamine/12.5 mg/kg xylazine anesthesia injection. Mice were laid on a clean paper and then, a vertical midline incision with scissors cut from the neck to pubis and opens the peritoneum. Then, the whole liver and kidney tissue were taken and transferred to organ tube containing the 10% formalin for preservation and transportation. Then, in gross room liver and kidney tissue were excised from specific part of each collected sample by histopathology laboratory technician and transferred into tissue processing cassette. The tissues were processed overnight in open tissue processors. The both organ tissues in the cassette rotates overnight over different reagent containing jars starting from the 10% formalin which completely immerses the tissues for the purpose of fixation. Then, tissues were dehydrated in a series of an increased ordered ethanol concentration of (70, 80, 90, and 100%). Xylene was used to remove ethanol from the tissues and replace it with fluid which is soluble with paraffin. The tissues were embedded in paraffin wax with the help of electro-thermal wax dispenser to form tissue blocks in squared metallic plates block molds. The blocks were then labeled, and placed in a refrigerator until sectioned. Microtome was used for sectioning of tissue blocks manually. The paraffin blocks having tissues were put in the rotary microtome. The ribbon of sections were carefully picked from the knife by a blunt forceps to float in a water bath of 40 to remove folds in the sections.

Unfolded sections were picked by clean microscope glass slides and were placed in an oven maintained at a temperature of 56 for 15 min for proper drying and better adhesion. The tissue sections were then cooled, dried and stained. The paraffin wax was removed from the tissue sections using xylene. The sections were then immersed in a series of descending alcohol concentration (100, 95, 70, and 40%) to remove xylene after which distilled water was used to hydrate the tissue. The hydrated sections were immersed in hematoxylin for 3–5 min with an eosin counterstained and agitated with acid alcohol to prevent over staining. Sections were immersed in a mixture of sodium 30 bicarbonate, ethanol and distilled water to give blue color to the nucleus. Then it was immersed in 95% alcohol and eosin to give pink color to the cytoplasm. Finally, tissue sections were dehydrated in 95% alcohol, cleared in xylene and mounted by adding a drop of DPX (Dibutyl Phthalate in Xylene) mounting medium on the section to cover the microscopic glass with cover glass and to increase the refractive index of the tissue under light microscope.

### Data analysis

The data entered to the Epidata version 3.1 and exported to statistical package for social science (SPSS) version 25 for analysis. Data expressed as mean ± SEM. One-way ANOVA done to determine statistical differences among all groups of the study. This followed by Tukey *post hoc* test using SPSS software version 25 and *p* < 0.05 considered as statistically significant. The results presented by tables and figures.

### Ethical considerations

The research conducted after getting an ethical approval letter from the Jimma University Institutional Review Board with a reference No. of IHRPGD/714/2020.

## Results

### Preliminary phytochemical screening

The preliminary phytochemical screening of *L. sativum* seed extracts using different solvents revealed the status of phytochemical constituents such as alkaloid, flavonoid, phenol, steroid, saponin, and quinone using different organic solvent extract as shown in Table 2.

**Table 2:** Preliminary phytochemical screening of *Lepidium sativum* seed.

Phytochemical constituents	Status			
	ME	HE	CE	AE
Alkaloid	+	+	+	+
Terpenoid	−	−	+	+
Flavonoid	+	−	−	+
Phenol	−	−	−	−
Steroid	+	−	−	−
Saponin	+	−	−	−
Quinone	+	−	+	+

The letter Indicate: ME, methanolic extract; HE, hexane extract; CE, chloroform extract; AE, acetone extract. (−) = Absent, (+) = Present.

### Percentage yield of *Lepidium sativum* L. seed extract

The amount of crude extracts which was obtained from 400 g coarse powder of *L. sativum* L. seed was 51.06 g. Therefore, the percentage yield was calculated and given as:

% yield= $\;\frac{51.06}{400}\times 100$ =12.8% (weight (w)/weight (w)).

### Effect of MSELS extract on body weight of mice

**Figure 1:**
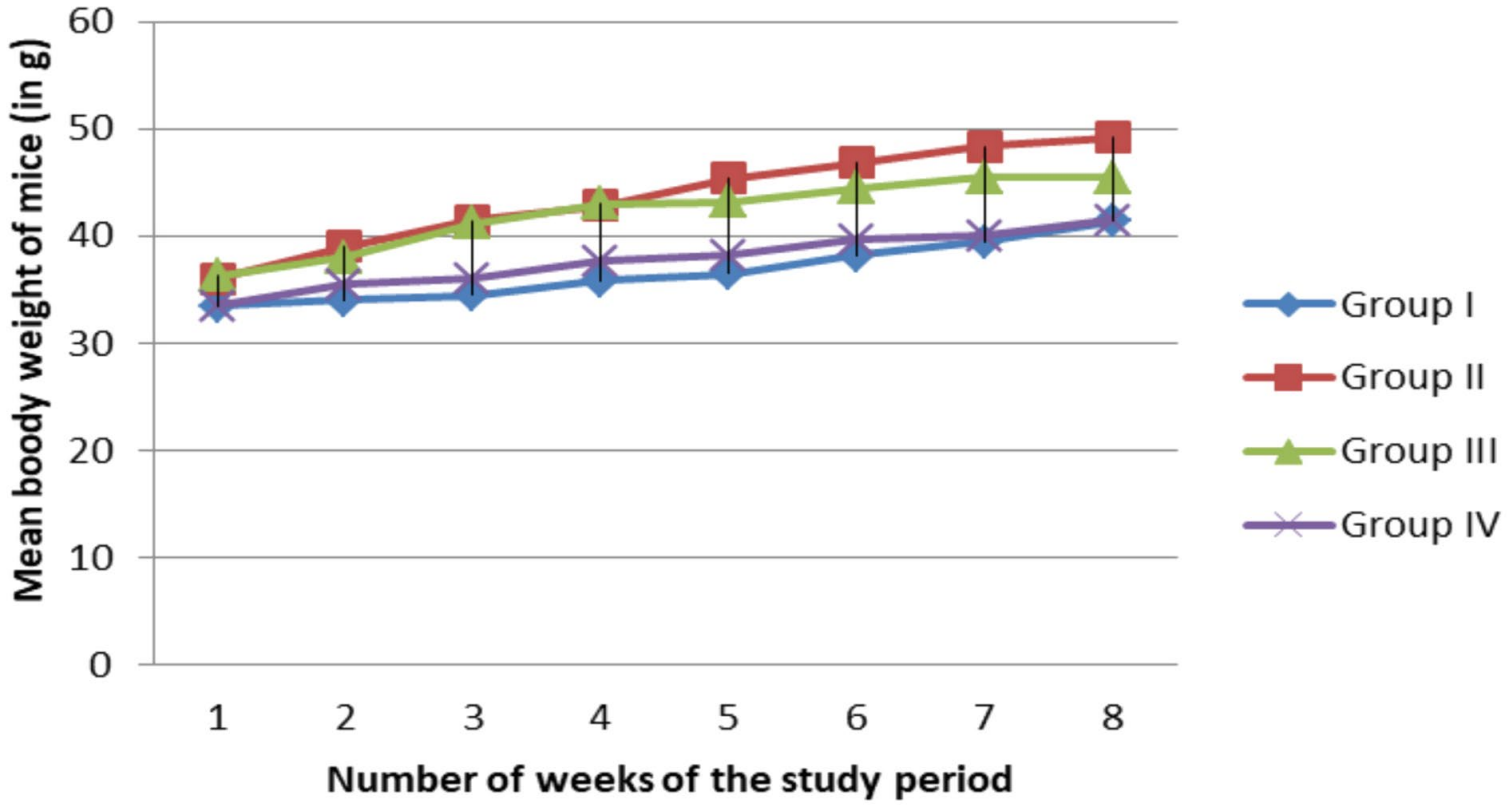
Effect of MSELS on the body weight at different weeks of the study period [expressed in terms of (M ± SEM)]. NB, body weight measured in gram (g, gram).

As shown in the Figure 1, all study groups showed non-significant (*p* < 0.05) increase in body weight at the end of each weeks even though the Group II mice showed more increasing of the body weight starting from week 4 than the remaining groups.

As shown in Table 3, the body weight change of the Group II that fed on the fried palm oil increased significantly when compared to the Group I. The body weight change of the Group III and IV, respectively, decreased significantly as compared to the Group II that fed on the fried palm oil. The liver weight of Group III and IV decreased when compared to the Group II even though only Group IV decreased significantly.

**Table 3:** Comparison of mean ± SEM value of body weight and liver weight among the four groups.

G. N	Body weight (g)			Liver weight(g)
	Initial body weight	Final body weight	Body weight change	
I	32.67 ± 0.67	41.33 ± 0.33	8.33 ± 0.49	2.04 ± 0.74
II	32.83 ± 0.48	49.17 ± 0.65	16.33 ± 0.95^a^	3.2 ± 0.88^a^
III	33.67 ± 0.71	45.50 ± 0.62	11.83 ± 0.87^b^	2.9 ± 0.62^a^
IV	32.17 ± 0.70	41.50 ± 0.85	9.50 ± 1.20^b^	2.3 ± 0.13^b^

The results expressed as mean ± SEM, (*n* = 6), *p* < 0.05, symbols represent statistical significance. ^a^Considered significantly different at *p* < 0.05 when compared with the group I. ^b^Considered significantly different at *p* < 0.05 when compared with the group II.

### Effect of MSELS on lipid profile

As shown in Table 4, the group II serum levels of TC, LDL-C, LDL-C/HDL-C and TG increased significantly whereas the HDL-C decreased significantly when compared to group I. In the MSELS treated groups, the serum level of TC, LDL-C, and LDL-C/HDL-C decreased but statistically significant reduction was observed in group-IV that were treated with 400 mg/kg/day of MSELS. However, the serum level of HDL-C was increased but statistically significant increment observed in group-IV whereas the serum level of TG reduced significantly both at 200 and 400 mg/kg/day doses of MSELS (Table 5).

**Table 4:** Comparison of the mean ± SEM value of lipid profile among the four groups of the male Swiss albino mice.

G	Serum lipid level (mg/dl)				
	TC	HDL-C	LDL-C	LDL-C/HDL-C	TG
I	100.83 ± 3.27	82.17 ± 3.84	13.83 ± 1.49	0.14 ± 0.01	116.00 ± 3.52
II	129.71 ± 4.53^a^	45.43 ± 3.97^𝑎^	27.14 ± 2.30^𝑎^	0.57 ± 0.05^𝑎^	151.57 ± 3.59^𝑎^
III	120.00 ± 3.75^a^	63.60 ± 4.69	20.00 ± 2.07	0.35 ± 0.034^𝑎𝑏^	136.40 ± 2.89^𝑎^
IV	108.83 ± 4.48^b^	68.83 ± 6.68^𝑏^	16.83 ± 1.47^𝑏^	0.28 ± 0.06^𝑏^	125.33 ± 7.16^𝑏^

The results expressed as Mean ± SEM, *P* < 0.05: symbols represent statistical significance. ^a^Considered significantly different at *p* < 0.05 when compared with the Group I. ^b^Considered significantly different at *p* < 0.05 when compared with the Group II.

**Table 5:** Comparison of the mean ± SEM value of kidney function tests among the four groups of male Swiss albino mice.

Group	Kidney function tests (mg/dl)		
	Creatinine	BUN	Uric acid
I	0.65 ± 0.01	37.32 ± 5.58	6.09 ± 0.35
II	0.97 ± 0.06^𝑎^	49.14 ± 5.15	11.83 ± 0.54^𝑎^
III	0.61 ± 0.63^𝑏^	42.78 ± 3.55	10.06 ± 0.31^𝑎𝑏^
IV	0.59 ± 0.74^𝑏^	38.44 ± 4.15	6.58 ± 0.31^𝑏^

The results expressed as mean ± SEM. Symbols represent statistical significance. ^**a**^Considered significantly different at *p* < 0.05 when compared with the normal control group. ^b^Considered significantly different at *p* < 0.05 when compared with the group II.

### Histopathology

**Figure 2:**
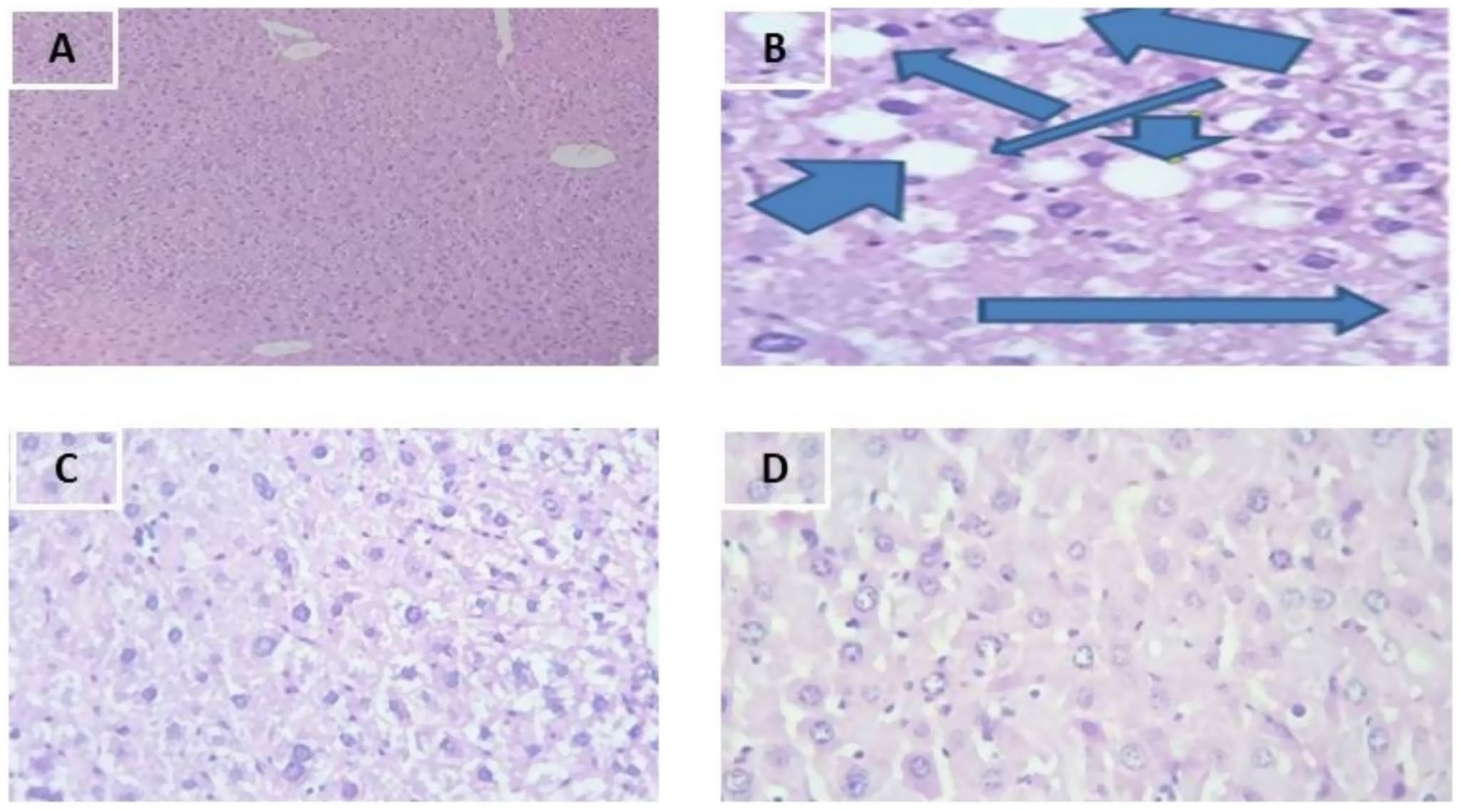
Photomicrographs of 40×, Hematoxylin (H) and Eosin (E) stained liver sections of mice. **(A)** Group I, **(B)** Group II -Liver sections present severe degenerative changes in hepatocytes including fatty changes (arrows), **(C)** Group III, **(D)** Group IV.

As shown in Figure 2, in addition to the body weight, liver weight and biochemical result of the lipid profile, the liver histopathology indicates the effect of the plant extract on the dyslipidemia induced by fried palm oil. As shown in the following figures, the group II showed induced dyslipidemia especially through increased triglycerides in the liver. The Group III and IV showed ameliorative effects that are range from moderate to almost near to the normal group in architecture of the liver.

### Kidney histopathology

**Figure 3:**
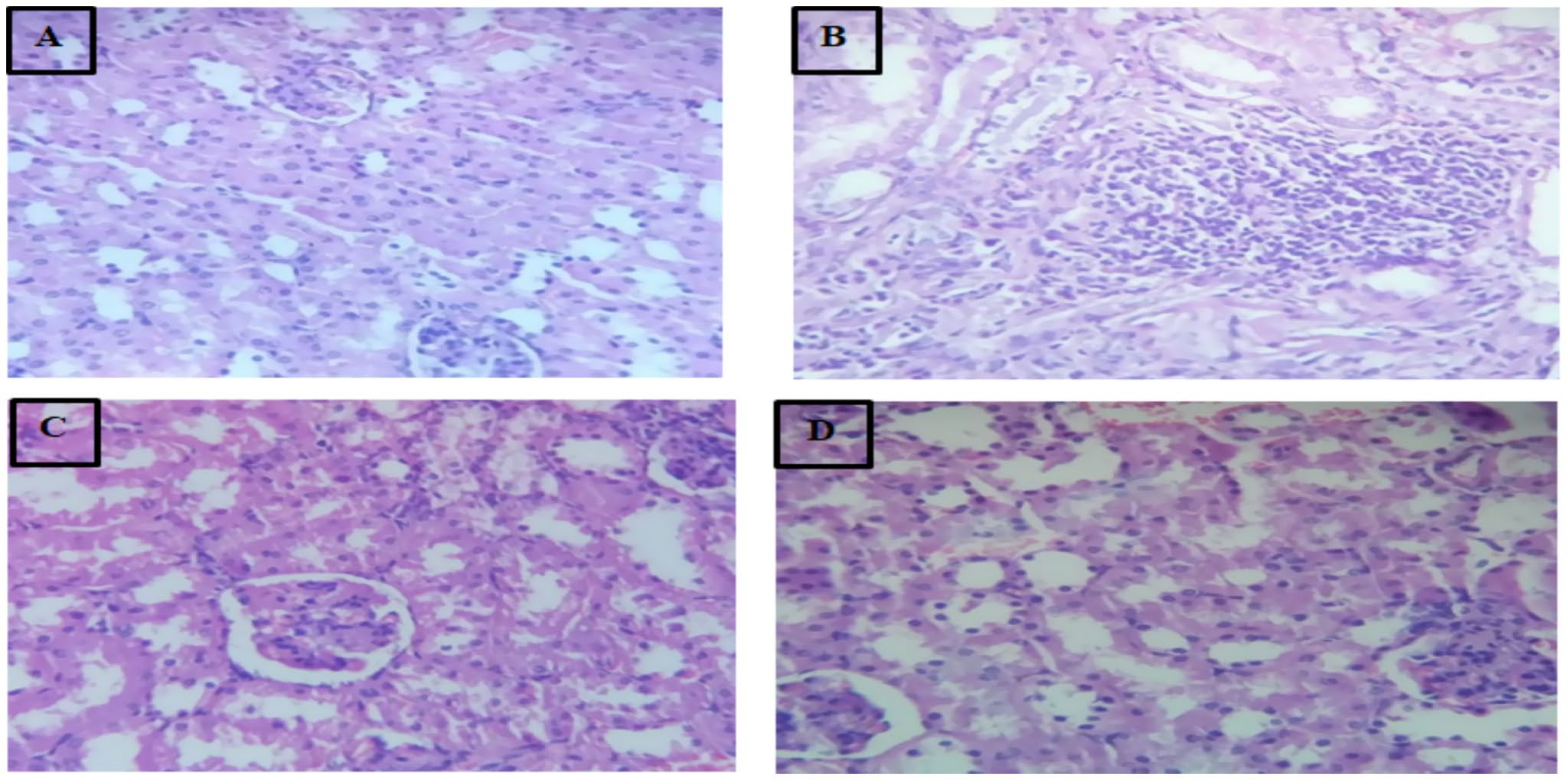
Photomicrograph of 40×, Hematoxylin (H) and Eosin (E) stain kidney tissues of mice. **(A)** Group-I; **(B)** Group-II; **(C)** Group-III; **(D)** Group-IV.

Photomicrograph of 40×, Hematoxylin (H) and Eosin (E) stain kidney tissues of mice is shown in Figure 3.

## Discussion

### Effects of MSELS on lipid biochemical parameters

Frying degrades the important bioactive ingredients of the oils and results in the formation of many harmful products for health. However, the practice of reusing oil during frying was still widely practiced in the community. As the long-term consumption of recycled frying oils may promote the derangement to lipid profile and kidney, it is important to look for the ways amenable to the daily diet to mitigate the associated adverse health effect of it (36, 37).

In the present study, the group II serum TC, LDL-C, LDL-C/HDL-C and TG values increased almost significantly whereas decreased in HDL-C when compared to group I serum values. This study findings were in line with the previous study findings of Famurewa et al. (32), Okwari et al. (33), and Abeer et al. (38). Oxidation reaction during deep-frying causes changes in fatty acid configuration from the *cis* isomer to the *trans* isomer. The increase in the serum TC, LDL-C, LDL-C/HDL-C and TG as well as decrement in HDL-C in the present study might be due to the increased *trans*-fat, saturated fatty acid and availability of free fatty acid content in the fried palm oil (38, 39).

However, this study finding was inconsistent with the study findings of Badr et al. (39) and El-Zawahry et al. (40). Even though the exact reasons for variations are not clear, the variation might be due to the difference in the process of frying, the temperature of frying, time of frying, fried food item and types of animals used. In the group that administered MSELS the serum TC, LDL-C, LDL-C/HDL-C and TG values were decreased even though it showed more significant decrement at dose of 400 mg/kg/day whereas the HDL-C showed increment when compared to group II that fed on fried palm oil only. This study finding was in harmony with the study results of El-Zawahry et al. (40), Chauhan et al. (41), Althnaian (42), and Bigoniya et al. (43). This might be attributed due to flavonoid (naringenin) inhibition of cholesterol biosynthesis through inhibition of hydroxymethylglutaryl coenzyme A (HMG-CoA) reductase, the rate-limiting enzyme which mediates the major step in cholesterol biosynthesis (40). It might be also due to the saponin constituents of *L. sativum* seed that form insoluble saponin cholesterol complexes within gastrointestinal tract which prevents absorption of cholesterol like saponin of the *Thymus vulgaris* (44). The possible mechanism for the decreased LDL-C might be due to the flavonoid (kaempferol) constituents of seed on inducing the increased low-density lipoprotein receptor gene expression (45).

The decreased TG might be due to *L. sativum* seed secondary metabolites inhibition of absorption and enhanced excretion of lipids. Besides, it might be due to the better efficiency of flavonoid constituents (apigenin) of the extract toward increasing HDL-C. The flavonoid (apigenin) constituents enhance the expression of the ABCA1 gene and this facilitate the cholesterol efflux from the macrophage and in turn increase the HDL-C in the circulation (46). However, the present study findings contradict with Althnaian (42) study report. The exact causes for these variations are unclear. However, it may be due to the variations in the mode of *L. sativum* seed administration, dose of MSELS and variation in study period. In addition to the lipid panel, as shown in Table 3, the liver weight of group IV decreased significantly when compared with Group II. This might be due to restored activity of hepatocyte normal function by the action of different flavonoid type in the seed extract which enhances activation fat oxidation pathway.

### Effects of MSELS on kidney function tests

In present study, Group II serum creatinine and uric acid values increased significantly whereas the serum BUN value increased non-significantly when compared with Group I. This study finding was in line with Amsalu et al. (19), Badr et al. (39), and Amany et al. (44). This might be resulted due to increased oxidative stress, which results from repeated heating of palm oil. This indirectly or directly induces deterioration of renal function through excessive reactive oxygen species (ROS) such as hydroperoxides, which lead to cellular damage by reacting with various biomolecules such as proteins, nucleic acids, or lipids (20). The MSELS showed decrement in kidney function tests in Group III and IV when compared to the Group II. Accordingly, the serum creatinine and uric acid was significantly decreased in MSELS treated Group III and IV when compared to Group II. The serum creatinine showed significant decrement at both 200 and 400 mg/kg of MSELS whereas serum uric acid showed significant decrement at only 400 mg/kg of MSELS. This study’s results were in agreement with the study findings in Balgoon (47). This might be due to the phytoconstituents of the LS extract that restore the structure and function of the kidney and cause an increased glomerular filtration rate while also acting as a diuretic agent. In contrast, the current study finding contradicts the previous study finding (42). The clear cause of variation of the current study findings from the earlier not known. However, the variation might be due to the difference in the mode of *L. sativum* administration. In the current study, the extract form that was administered to mice, whereas in the previous one it was in the form of powder.

### Liver histopathology

As shown in the Figure 2B, the liver architecture of group II mice showed liver parenchyma with severe vacuolar and fatty change showing ring appearance. This study results were in agreement with the study of El-Zawahry et al. 40. In addition to reduction in liver weight, the simultaneous treatment of mice with MSELS in-group III and IV showed improvements to the damaged liver histopathology. As shown in Figure 2C, at 200 mg/kg/day of MSELS was showed mild vacuolar degeneration whereas at 400 mg/kg/day of MSELS (48) as shown in Figure 2D showed almost normal hepatic parenchyma composed of central veins and portal tracts with portal veins which approves the restoring effect of MSELS due to its combined bioactive components. The current study findings were in line with study findings of Zamzami et al. (49).

### Kidney histopathology

In the deep fried palm oil only fed group II the image of microscopic slide showed that the kidney tissue undergoes severe histopathological alterations such as fibrosis, lymphocytic infiltrations, local inflammations, tubular necrosis and inflammatory infiltration. The present study finding was in agree with previous study of Amany et al. (44). This might be due to the production of toxic products that generated during repeatedly frying of palm oil that induce oxidative stress and damage to renal tissue. The experimental groups that treated with MSELS showed improvement or restoration of the damaged renal architecture almost toward normal. This study’s results are in line with the study findings of Balgoon (47). This might be due to the mitigation of the kidney tissue by MSELS. The mitigation effect of MSELS attributed to its different bioactive ingredients, which have the ability to reduce oxidative stress, inflammation, and apoptosis in the renal tissue (50).

### Limitations of the study

- Analysis of the products which formed during frying oils was not performed.
- Only two different doses of seed extract were investigated to assess the effect of the *Lepidium sativum L.* extract.
- Phytochemical analysis of the specific marker compound and their content of the *Lepidium sativum L.* extract was not performed.
- The comparison of *Lepidium sativum L.* seed extract with the standard drugs having lipid lowering and hepatoprotective effects was not conducted.

## Conclusion

Based on the above current study findings, mainly observed from liver weight, lipid profile, kidney function tests, kidney and liver histopathology the MSELS have a prominent ameliorating effect on fried palm oil induced alteration of the lipid profile (dyslipidemia) as well as kidney function damage on male Swiss albino mice. Therefore, study revealed that as the MSELS have an antidyslipidemic and nephroprotective activity on the damaged renal tissue following treatment with deep fried palm oil. The protective effect of the MSELS might be due to the combined effect of the different bioactive compounds in the extract. Therefore, the MSELS might be contain promising chemical compound on different chronic disease which need further study for isolation and evaluating the effect of specific compound on specific disease.

## Data Availability

The original contributions presented in the study are included in the article/supplementary material, further inquiries can be directed to the corresponding author.
